# Late Maturation of Adult-Born Neurons in the Temporal Dentate Gyrus

**DOI:** 10.1371/journal.pone.0048757

**Published:** 2012-11-07

**Authors:** Jason S. Snyder, Sarah C. Ferrante, Heather A. Cameron

**Affiliations:** Unit on Neuroplasticity, National Institute of Mental Health, National Institutes of Health, Bethesda, Maryland, United States of America; Institut National de la Recherche Agronomique-CNRS UMR6175, France

## Abstract

Hippocampal function varies along its septotemporal axis, with the septal (dorsal) pole more frequently involved in spatial learning and memory and the temporal (ventral) pole playing a greater role in emotional behaviors. One feature that varies across these subregions is adult neurogenesis. New neurons are more numerous in the septal hippocampus but are more active in the temporal hippocampus during water maze training. However, many other aspects of adult neurogenesis remain unexplored in the context of septal versus temporal subregions. In addition, the dentate gyrus contains another functionally important anatomical division along the transverse axis, with the suprapyramidal blade showing greater experience-related activity than the infrapyramidal blade. Here we ask whether new neurons differ in their rates of survival and maturation along the septotemporal and transverse axes. We found that neurogenesis is initially higher in the infrapyramidal than suprapyramidal blade, but these cells are less likely to survive, resulting in similar densities of neurons in the two blades by four weeks. Across the septotemporal axis, neurogenesis was higher in septal than temporal pole, while the survival rate of new neurons did not differ. Maturation was assessed by immunostaining for the neuronal marker, NeuN, which increases in expression level with maturation, and for the immediate-early gene, Arc, which suggests a neuron is capable of undergoing activity-dependent synaptic plasticity. Maturation occurred approximately 1–2 weeks earlier in the septal pole than in the temporal pole. This suggests that septal neurons may contribute to function sooner; however, the prolonged maturation of new temporal neurons may endow them with a longer window of plasticity during which their functions could be distinct from those of the mature granule cell population. These data point to subregional differences in new neuron maturation and suggest that changes in neurogenesis could alter different hippocampus-dependent behaviors with different time courses.

## Introduction

It is becoming increasingly clear that the hippocampus is not a homogeneous structure but instead has different properties and functions associated with its septal and temporal subregions [1], [2]. Generally, lesions directed at the septal pole adversely affect spatial learning and memory, whereas lesions of the temporal pole have been found to be anxiolytic [3], [4]. Anatomically, this dissociation may be mediated by differential connectivity with the entorhinal cortex [5], [6], amygdala and hypothalamus [7], [8]. While septal and temporal hippocampus do not have mutually exclusive roles in spatial processing and emotional behaviors, their functions do appear distinct and complementary. For example, spatial information is represented at different resolutions along the septotemporal axis [9], and septal and temporal subregions are required for distinct aspects of spatial navigational behavior [10], [11], [12], [13]. Heterogeneity is also seen across the transverse axis of the dentate gyrus, that is, between the suprapyramidal and infrapyramidal blades. Although there are no studies addressing functional differences between suprapyramidal and infrapyramidal granule cells at the behavioral level, there are well-described differences between granule cells in the two blades in the size of the dendritic tree, ratio of inhibitory interneurons, connectivity, and experience-induced activation [14], [15], [16], [17], [18], [19], [20]. Due to this heterogeneity, understanding the function of the hippocampus requires subregion-specific investigations.

Ongoing production of granule neurons in adulthood occurs throughout the length of the dentate gyrus and in both blades [21], [22], [23], [24]. Very few studies have addressed the regulation or relative rates of adult neurogenesis in the two blades. Increased cell proliferation and neurogenesis have been observed in the infrapyramidal blade relative to the suprapyramidal blade in normal rats [21], [25]. A study of seizure effects on neurogenesis found that although seizures increased cell proliferation in both blades, extra neurons were retained at longer time points only in the infrapyramidal blade, demonstrating differential survival of new neurons in the two blades under these conditions [26]. However, although differences in connectivity suggest activity-dependent differences in maturation across blades, this has not been examined.

The potential functional importance of differences between septal and temporal neurogenesis has been better appreciated [27], but actual differences are only beginning to be characterized. Under control conditions, more neurogenesis occurs in the septal than temporal dentate gyrus (DG) of both rats [21] and mice [22]. Interestingly, both antidepressants and oxytocin increase granule cell precursor proliferation primarily within the temporal DG [24], [28], while stress inhibits proliferation more strongly in this same region [29], consistent with an important relationship between new neurons, stress responses and depressive behavior [30], [31], [32]. No studies have compared the survival of new neurons across septotemporal subregions. The lack of discrete boundaries or identifying features of septal and temporal subregions is likely to be at least partly responsible for the paucity of septotemporal studies of adult neurogenesis. Stereological cell counting methods, which are now the gold standard for quantitative cell counting, are designed for regions of interest with clear, identifiable boundaries [33], [34], which the septotemporal domains of the hippocampus lack. The curvature of the hippocampus further complicates subregional analyses in any of the standard anatomical planes.

The morphological and physiological maturation process of adult-born granule cells has been described in several studies [35], [36], [37], [38], [39], [40], but a study by Piatti et al. is the only one to date to compare septotemporal (rostrocaudal) regions [23]. These authors found that electrophysiological properties of new neurons in the septal DG mature faster than those in the temporal DG and proposed that this could be due to increased levels of network activity [23]. Since neuronal activity is often linked to cell survival, these findings predict that survival of new neurons may be different between the septal and temporal hippocampus. Accelerated electrophysiological maturation in the septal hippocampus also suggests that these neurons should be integrated into functional circuits at a younger age. The immediate early gene (IEG) Arc is well-characterized as a critical effector IEG required for learning and plasticity [41], so its expression in vivo following maximal stimulation can be used to gauge whether or not a neuron is synaptically integrated and capable of contributing to hippocampal function [42], [43]. We find that neuronal survival differs across blades but not along the septotemporal axis. In contrast, maturation rate does not differ across blades but is dependent on septotemporal location, with septally-located neurons gaining the ability to express Arc and strong NeuN more rapidly than those in the temporal DG. These data suggest that the timing of functional significance of neurogenesis may differ along the septotemporal axis.

## Methods

### Animals and Treatments

A total of 25 adult male Sprague Dawley rats (Charles River) were treated as previously described [39]. Sexually naïve rats were received at 8 weeks of age and acclimated to the animal facility for 1 week prior to any manipulation. For the duration of the experiment, rats were housed 2 per cage in ventilated racks with food and water available *ad libitum* and a 12∶12 h light:dark schedule with lights on at 6∶00 A.M. All procedures followed the Institute of Laboratory Animal Research guidelines and were approved by the Animal Care and Use Committee of the National Institute of Mental Health.

**Figure 1:**
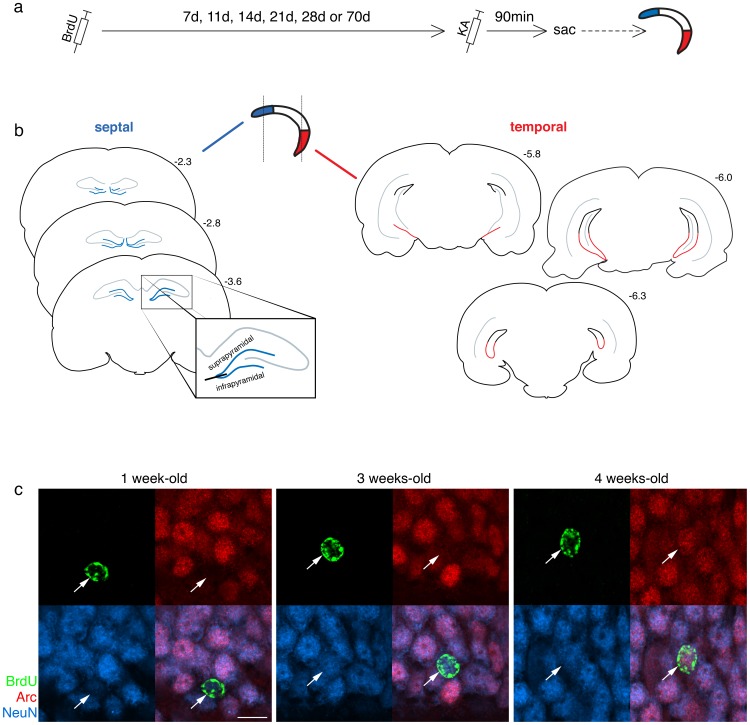
Experimental design. a) Experimental timeline. Rats received a single injection of BrdU 7, 11, 14, 21, 28 or 70 days prior to injection with kainic acid (KA) to induce seizures, which upregulate Arc expression. Rats were perfused 90 min after seizure induction for septotemporal DG histological analyses. b) Coronal sections showing anatomical boundaries used for delineating septal and temporal subregions (adapted from [46]). In rostral sections, the entire DG is septal (illustrated in blue). In caudal sections, the ventral half of the DG was defined as temporal (illustrated in red). Numbers indicate anterior-posterior position in mm, relative to Bregma. c) Representative confocal images of BrdU+ adult-born neurons (arrows) immunostained for Arc and NeuN. Arc expression was absent in 1-week-old cells, moderate in 3-week-old cells, and strong in 4-week-old cells. NeuN expression was observed in all cells but became progressively stronger with cell age. Scale bar, 10 µm.

Rats were given a single injection of bromodeoxyuridine (BrdU; Roche; 10 mg/ml in saline with 0.007 N NaOH, i.p.) and perfused either 7, 11, 14, 21, 28, or 70 d after BrdU injection (Fig. 1; n = 3–5 per group). On the day of perfusion, kainic acid (15 mg/kg; i.p.; Tocris Bioscience) was given to strongly activate granule cells and induce IEG expression throughout the DG, as previously described [39]. Within 60–90 min of kainic acid injection, rats developed stage 5 seizures, characterized by episodes of rearing and falling [44], which were used to monitor neuronal activation. Convulsions were stopped by injection of the GABA agonist sodium pentobarbital (50 mg/kg; i.p.) 30 min after the onset of stage 5 seizure activity. Rats were perfused 60 minutes after sodium pentobarbital injection (i.e., 90 min after stage 5 seizure onset). Two rats that did not have seizures were excluded.

### Histological Methods

Rats were perfused with 4% paraformaldehyde in phosphate buffered saline (PBS; pH 7.4). Brains remained in fixative overnight after which they were transferred to a 10% glycerol solution for 24 h and then a 20% glycerol solution for 48 h before being processed. Brains were sectioned coronally on a freezing microtome at 40 µm thickness. Some sections from these brains were used for other studies [39]. For BrdU counts, a 1 in 12 series of sections throughout the entire DG were mounted onto slides, heated in citric acid (0.1 M, pH 6.0) for 10 min for antigen retrieval, permeabilized with trypsin for 10 min, and denatured in 2 N HCl for 30 min. Sections were then incubated with mouse anti-BrdU antibody (1∶100; BD Biosciences) at 4°C overnight followed by biotinylated goat anti-mouse IgG (1∶200; Sigma) at room temperature for 1 h. BrdU was then visualized using an avidin-biotin-horseradish peroxidase kit (Vector Laboratories) and cobalt-enhanced DAB (Sigma Fast tablets). Slides were then counterstained with cresyl violet acetate and coverslipped with Permount.

The Arc and NeuN expression profile of BrdU+ cells was determined using immunohistochemical triple labeling with fluorescent detection. Free-floating sections were heated at 90°C in citric acid (0.1 M, pH 6.0) for 25 min to eliminate non-specific staining of blood vessels and expose antigens, then treated with 2 N HCl for 1 h. Sections were then incubated for 3 d at 4°C in PBS containing 0.5% Triton-X, 3% donkey serum and all of the following primary antibodies: rat anti-BrdU antibody at 1∶500 (Accurate, OBT0030), mouse anti-NeuN (anti-Fox-3) at 1∶250 (Millipore, MAB377), and rabbit anti-Arc at 1∶4000 (Synaptic Systems, 156 003). Sections were subsequently incubated for 90 minutes at room temperature in donkey anti-rat Alexa488, donkey anti-mouse Alexa647, and donkey anti-rabbit Alexa555 antibodies (Invitrogen), all diluted 1∶250 in PBS. Sections were then mounted onto slides and coverslipped with Prolong Gold (Invitrogen).

### Histological Data Analysis

DAB-labeled BrdU+ cells in the granule cell layer were counted bilaterally in a 1 in 12 series of sections, using a 40× objective as previously described [39]. Analyses were limited to the septal and temporal poles of the DG. Due to the curvature of the hippocampus, with the septal end extending along the anterior-posterior (rostral-caudal) axis and the temporal end extending along the dorsal-ventral axis, no single anatomic plane can be used to accurately define septal and temporal hippocampal subregions [45]. Therefore, the septal and temporal poles were defined in our coronal sections as follows: septal analyses began at 3.3 mm posterior to Bregma [46] and extended rostrally to the anterior end of the DG. Temporal analyses began at 4.5 mm dorsal to the interaural line and extended to include all ventral portions of the DG (Fig. 1). For practical purposes, in coronal sections, the temporal DG was delineated by bisecting posterior sections (−5.2 to −7.0 mm relative to Bregma) and analyzing only the ventral half. All BrdU+ cells located in the granule cell layer or within 20 µm of the inner border of the granule cell layer (in the subgranular zone) were counted. Cells were also classified according to their position with the infrapyramidal vs. suprapyramidal blades. Blade analyses were limited to the septal DG since the two blades could not be reliably discriminated in the caudalmost sections. BrdU+ cells were counted as they came into focus in order to avoid oversampling cell fragments that appear in multiple sections. One rat was excluded due to an absence of BrdU immunostaining.

Count data were converted to densities, since volumes of septal and temporal poles were not necessarily equivalent and could not be biologically determined due to the lack of detectable changes along this axis. Cell density measurements were obtained by dividing the number of BrdU+ cells counted in each subregion by the total volume of the granule cell layer in that subregion in the analyzed sections. Volume measurements were obtained as previously described [19]. Briefly, the granule cell layer was traced on cresyl violet stained sections, using Stereoinvestigator software (Microbrightfield) and a 4×objective. The resulting cross-sectional area was multiplied by the section thickness (40 µm).

Arc and NeuN expression was examined in 25–30 BrdU+ cells from each of the septal and temporal poles (as defined above) from each animal, sampled from both the infrapyramidal and suprapyramidal, using a 60× oil-immersion lens (numerical aperture, 1.25). Due to small numbers of BrdU+ cells, multiple evenly spaced sections throughout the temporal DG were analyzed. In the septal DG, sampling was slightly more restricted due to the higher number of BrdU+ cells present. Analyses began at approximately −2.8 mm AP and extended rostrocaudally when too few cells were present. To objectively quantify graded expression of Arc and NeuN in BrdU+ cells, fluorescence intensity was measured and compared to background levels within each field, as previously described [39], [42]. Fluorescence was measured at the center of the cell body in order to consistently measure the strongest signal and therefore only BrdU+ cells that were present in their entirety were examined. Cells were considered Arc-positive when Arc expression was ≥1.5×background, a threshold that captured the minimal amount of Arc expression that could be considered unambiguously positive by eye. BrdU+ cells were considered strongly NeuN-positive when expression exceeded 6×background, which excluded weakly NeuN+ immature neurons [39]. For both markers, expression levels are presented as the percentage of BrdU+ cells that met these criteria.

### Statistics

Statistical comparisons were made using two-way ANOVA, with septotemporal region as a repeated measure (Graphpad Prism software). Significance was set at *p*<0.05. Bonferroni *post hoc* tests were used to compare septal and temporal cohorts of neurons of a given age.

## Results

### Cell Survival in Septal Versus Temporal and Infrapyramidal Versus Suprapyramidal Subregions

**Figure 2:**
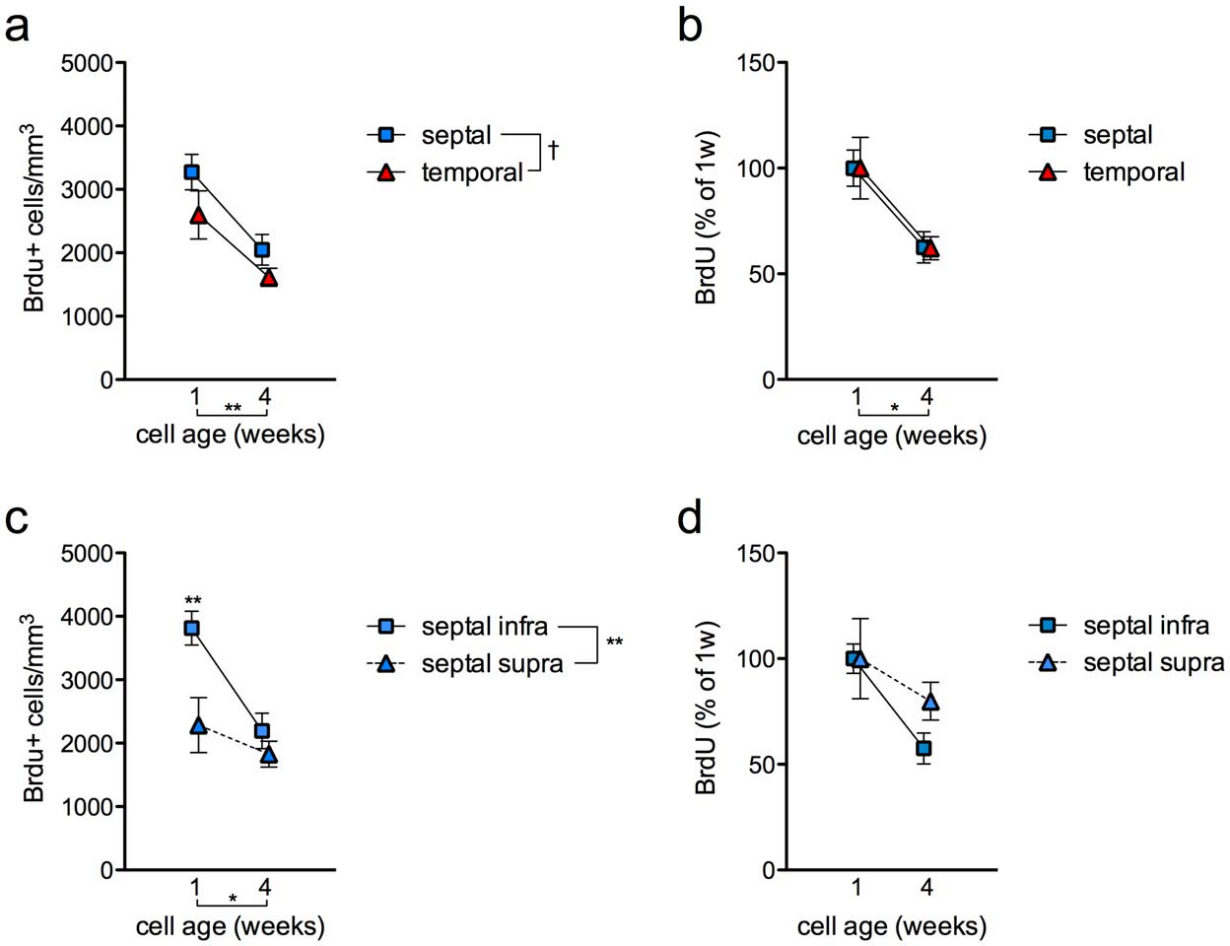
Subregional survival of BrdU+ cells. a) The density of BrdU+ cells decreased similarly in the septal and temporal DG from 1 w to 4 w post-BrdU injection. There was a trend for less overall neurogenesis in the temporal DG (effect of cell age F_1,7_ = 13, P<0.01; effect of septotemporal subregion F_1,7_ = 4, P = 0.09; interaction F_1,7_ = 0.2, P = 0.7). b) Normalizing each region’s BrdU+ cell density to the 1 w value, a similar proportion of cells were lost between 1 w and 4 w in the septal and temporal DG (effect of cell age F_1,7_ = 12, P<0.05; effect of septotemporal subregion F_1,7_<0.01, P = 1; interaction F_1,7_<0.01, P = 1). c) In the septal DG, significantly more BrdU+ cells were initially added to the infrapyramidal blade, but by 4 weeks similar densities of cells were present in the two blades (effect of cell age F_1,7_ = 6, P<0.05; effect of blade F_1,7_ = 22, P<0.01; interaction F_1,7_ = 8, P<0.05; post hoc P<0.01 vs. septal supra at 1 week and septal infra at 4 weeks). d) Normalizing to BrdU+ cell densities at 1 week, in the septal DG, 80% of BrdU+ cells in the suprapyramidal blade survived to 4 weeks but only 58% in the infrapyramidal blade (not significant). ^†^P<0.1, *P<0.05,**P<0.01.

To explore potential septotemporal differences in cell survival, BrdU+ cell densities were compared at the 1 w and 4 w time points after BrdU injection (n = 4–5 per group). There was a trend for lower BrdU+ cell density in the temporal DG (Fig. 2a), consistent with previous findings showing reduced neurogenesis in the temporal DG as compared to the septal DG [21], [22]. There were fewer BrdU+ cells present at 4 w after BrdU injection compared with 1 w after injection, as expected due to cell death during this period [39], [47], [48]. Normalizing BrdU+ cell density to the 1w values to look at survival rates revealed that virtually identical proportions of cells survive in the septal and temporal DG (63% and 62%, respectively; Fig. 2b). When analyzed by blade, we found that the density of 1-week-old BrdU+ cells in the septal dentate gyrus was greater in the infrapyramidal blade than the suprapyramidal blade (Fig. 2c). However, a greater proportion of cells was subsequently lost from the infrapyramidal blade, indicating a lower survival rate and resulting in similar densities of 4-week-old cells (Fig. 2c, 2d).

### Expression of the Immediate-early Gene Arc in Septal Versus Temporal and Infrapyramidal Versus Suprapyramidal Subregions

**Figure 3:**
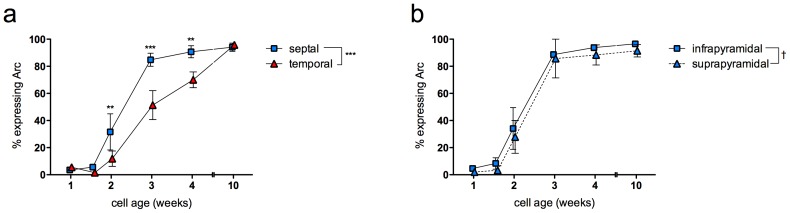
Timecourse of Arc expression across subregions. a) Expression of Arc in adult-born granule neurons was age-dependent and occurred earlier in the septal DG than in the temporal DG (effect of cell age F_5,19_ = 53, P<0.0001; effect of septotemporal subregion F_1,19_ = 35, P<0.0001; interaction F_5,19_ = 7, P<0.001). b) There were no differences between blades in the septal DG (effect of cell age F_5,19_ = 33, P<0.0001; effect of blade F_1,19_ = 3, P<0.1; interaction F_5,19_ = 0.1, P = 1). ^†^P<0.1, **P<0.01, ***P<0.001 post hoc vs. temporal value at same time point.

Arc expression was examined in adult-generated neurons of various ages following kainate-induced seizures (n = 3–5 per group). We found that the proportion of cells expressing Arc was negligible at 7 and 11 days after BrdU, but this proportion increased thereafter, reaching maximal levels by 10 weeks of age, consistent with our previous data using the IEGs zif268 and Fos [39]. Comparing anatomical subregions, we found that the ability of adult-born neurons to express Arc is accelerated in the septal DG as compared to the temporal DG (Fig. 3). A significantly greater proportion of septal adult-born neurons expressed Arc at 2, 3, and 4 weeks of age. However, by 10 weeks of age, the amount of Arc expression is the same in the two regions, suggesting that earlier differences in Arc reflect differences in maturation rather than differences in kainate-induced Arc expression in the overall granule cell population in the two regions. Over a span of 10 days, 80% of new neurons in the septal portion of the DG become capable of expressing Arc (5% at 11 days-old, 85% at 21 days-old). In contrast, only 50% of new neurons in the temporal DG begin to express Arc over this time period, and even at 4 weeks of age only 70% expressed Arc. To explore subregional differences along the transverse axis, the septal data were segregated by blade. The timecourse of Arc expression was similar in infrapyramidal and suprapyramidal blades of the septal DG (Fig. 3b).

### Septotemporal Expression of NeuN

**Figure 4:**
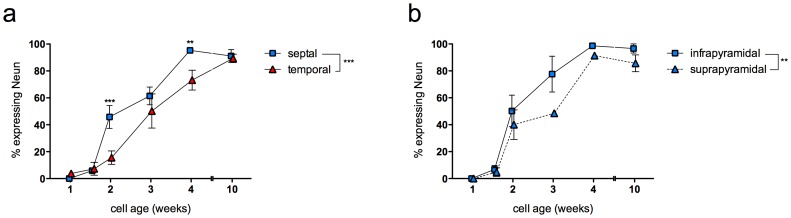
Timecourse of strong NeuN expression across subregions. a) Adult-born granule neurons increasingly expressed strong levels of NeuN with age and expressed NeuN earlier in the septal DG than in the temporal DG (effect of cell age F_5,19_ = 109, P<0.0001; effect of septotemporal subregion F_1,19_ = 18, P<0.001; interaction F_5,19_ = 7, P<0.01). b) Within the septal DG, overall NeuN expression was greater in the infrapyramidal blade (effect of cell age F_5,19_ = 84, P<0.0001; effect of blade F_1,19_ = 9, P<0.01; interaction F_5,19_ = 2, P = 0.2). **P<0.01, ***P<0.001, post hoc vs. temporal value at same time point.

NeuN is typically considered to be a marker of mature neurons, but it can be detected in all neurons by one week of age [39]. However, since NeuN shows graded expression depending on neuronal age, strong NeuN immunostaining is an accurate marker of neuronal maturity [39], [49]. In the same cells examined for Arc expression, we therefore quantified strong NeuN expression as an additional measure of cell maturity. A greater proportion of newborn neurons expressed NeuN in the septal than in the temporal DG, particularly at the 2- and 4-week time points (Fig 4a). These findings are consistent with the Arc data, providing further support for slower maturation of new granule cells in the temporal DG. There was also a significant difference in NeuN expression between the two blades, with more infrapyramidal neurons showing strong NeuN expression (Fig. 4b). However, since this difference was seen even in 10-week-old cells, and since Arc analysis shows no difference across blades, it seems likely that this difference in NeuN across blades reflects a difference in normal NeuN expression between the suprapyramidal and infrapyramidal granule cell populations rather than a difference in maturation.

## Discussion

### Septotemporal Location Effects on Maturation

Adult neurogenesis has been linked to both spatial memory- and emotion-related behaviors, which in turn have been linked to different hippocampal subregions. A better understanding of subregional differences in adult neurogenesis may therefore help elucidate the roles of new neurons in different types of hippocampus-dependent behavior. Here we report that while there are no differences in the survival rate of new granule cells across the septotemporal axis, there are pronounced differences in the time course of maturation, with new neurons maturing more than 1 week earlier in the septal DG than in the temporal DG (Table 1). The temporal delay in maturation was seen both in the proportion of young granule cells expressing Arc, an immediate-early gene related to firing behavior whose expression requires synaptic integration [50], [51], and in the proportion showing strong NeuN expression, a marker of mature granule neurons [39], [49].

**Table 1:** Summary of regional effects on new granule cell survival and maturation.^1^.

	New Neurons			
	1 wk	4 wks	Survival	Maturation
Septotemporal	sept > temp	sept > temp	sept = temp	sept > temp
Transverse	infra > supra	supra = infra	supra > infra	supra = infra

1 Sept, septal; temp, temporal; supra, suprapyramidal blade; infra, infrapyramidal blade. Symbols refer to *densities* of new neurons, *proportion* of neurons surviving, and *speed* of maturation.

A number of studies using retroviral labeling combined with electrophysiology or electron microscopy have provided a detailed characterization of the maturation time course of adult-born hippocampal neurons [36], [37], [38], [52], [53]. Immediate-early gene expression data provide a comparable maturation time course [35], [39], [40], [43], [54], [55]. The current data indicate that the time courses from these previous studies, which typically examined only the septal DG, likely underestimate the physiological and morphological maturation time for temporally-located new neurons. A recent study by Piatti et al. [23] found that adult-born temporal neurons acquire mature morphological and electrophysiological features more slowly than septal neurons. The agreement between our results and those of Piatti et al. suggests that this difference is robust across methods for assessing maturation as well as across species (mice and rats).

While a number of factors have been found to regulate new neuron survival and maturation, very little is known about subregion-specific regulation. Given that the septal and temporal hippocampus differ in gene expression [2], [56], levels of various neuromodulators [57], functional regulation by stress [58], and afferent inputs [6], [8] it is somewhat surprising that no septotemporal differences in survival were observed under control conditions in the current study. However these or other factors could potentially produce differential regulation of survival under different treatment conditions. Consistent with this possibility, it has recently been demonstrated that enriched environment preferentially increases neurogenesis within the dorsal DG whereas chronic stress and the antidepressant fluoxetine lead to decreases and increases, respectively, primarily within the ventral DG [29]. A potential subregion-specific role for these factors in maturation remains to be explored. Thus far, the only factor found to regulate maturation in a subregion-specific fashion is neuronal activity, which when increased via running accelerates maturation in the temporal DG and when reduced through overexpression of an inwardly-rectifying potassium channel delays maturation [23].

### Suprapyramidal-infrapyramidal Location Effects on Survival

In contrast to what was seen along the septotemporal axis, granule cells did not appear to mature at different rates across the suprapyramidal and infrapyramidal blades of the septal dentate gyrus. Arc expression in response to maximal stimulation showed nearly identical rates of expression across blades at all time points. The rate of strong NeuN expression was higher in the infrapyramidal blade; however, since this difference was still apparent in 10-week-old cells, it seems most likely that adult-born granule cells in this blade may have somewhat higher NeuN expression levels in general – an interpretation that is strengthened by the lack of evidence for a maturation effect in the Arc expression data. This possibility is supported by evidence that NeuN expression in the cortex is activity-dependent [59], although evidence from IEG studies suggests that the suprapyramidal blade is generally more active than the infrapyramidal blade [16], [17], [19].

The survival rate of new granule neurons varied across the transverse axis. The density of 1-week-old neurons was 67% greater in the infrapyramidal blade, but decreased survival in this blade resulted in similar densities of 4-week-old neurons in the two blades. Greater survival may be driven by greater neuronal activity in the suprapyramidal blade, i.e., a use-dependent mechanism, since it is well-established finding that the suprapyramidal blade shows greater increase in experience-dependent activity [16], [17], [21], [60], [61]. An alternative possibility, however, is that the lower activity in the infrapyramidal blade could lead to increased proliferation [62], reflected in the greater density of 1-week-old BrdU+ cells we observed and consistent with previous data indicating increased cell proliferation in the infrapyramidal blade than the suprapyramidal blade of the septal region in Sprague Dawley rats [25]. Reduced survival of these cells, then, could result from a homeostatic mechanism that aims to keep the final density of new cells equivalent, leading to similar densities of 4-week-old cells in the two blades. Although direct evidence for such a homeostatic mechanism is lacking, there are other examples of early increases in granule cell production that are lost due to increased cell death [42], [63], [64].

The lack of difference in 4-week-old neuron density across the two blades seen in the current study appears inconsistent with previous data from our lab showing a greater density of mature new neurons in the infrapyramidal than suprapyramidal blade [21]. Since the current study found greater neurogenesis at the earlier but not the later time point, one possibility is that the BrdU+ cells in the earlier study had not finished the maturation-dependent loss of cells from the infrapyramidal blade. However, this seems unlikely, since the cells in the previous study were also 4 weeks old and were in Long Evans rats, which show somewhat faster, rather than slower, granule cell maturation [39]. It is possible that differences in neurogenesis across the transverse axis reflect strain differences in blade heterogeneity. Another possibility is that different experiences – either the several days of handling or random changes in the housing environment – are responsible for this difference in neurogenesis across blades. The idea that variability in the relative rate of neurogenesis between blades may be highly dependent on experience and environmental factors fits with a recent study suggesting that the mode of neurogenesis differs across blades, but only in certain housing environments [65].

### Functional Implications of Septotemporal Differences in Arc Expression and Maturation

With time, new neurons in the temporal DG become physiologically similar to new septal DG neurons [23] and express high, equivalent levels of calbindin [23], NeuN and Arc (current study). It is currently unknown whether new neurons exert their effects during the late immature period of heightened plasticity or only when they have fully matured [66]. If the latter, the delayed maturation of temporal granule cells would suggest that anxiety-related behaviors may be affected approximately 1–2 weeks later than spatial behaviors when neurogenesis is enhanced or inhibited. However, if new neurons are behaviorally important during their highly plastic late-immature period, then temporal neurons may have a prolonged window of functional plasticity relative to septal new neurons. This could make them more effective than septal new neurons at associating events spread out in time, a suggested function of immature granule neurons [23], [67]. Additionally, an extended window of immaturity could offset the lower levels of temporal neurogenesis, giving the septal and temporal DG comparable numbers of immature plastic neurons at a given time. Our previous observation that neurons in the deepest layer of the temporal DG are highly active in learning situations [21], [61] could be explained by such an extended window of plasticity.

Adult-born neurons have been implicated in many behavioral functions of the hippocampus, including spatial processing, reference and working memory, anxiety and depression. Functional differentiation along the septotemporal axis has the potential to reconcile these seemingly disparate roles. Our data identify maturation state and ability to respond to input with expression of plasticity-related genes as factors that could confer distinct functions for new neurons in “septal” vs. “temporal” behaviors.

## References

[1] Bannerman DM, Rawlins JN, McHugh SB, Deacon RM, Yee BK, et al (2004) Regional dissociations within the hippocampus–memory and anxiety. Neurosci Biobehav Rev 28: 273–283. 15225971 10.1016/j.neubiorev.2004.03.004

[2] Fanselow MS, Dong HW (2010) Are the dorsal and ventral hippocampus functionally distinct structures? Neuron 65: 7–19. 20152109 10.1016/j.neuron.2009.11.031 PMC2822727

[3] Kjelstrup KG, Tuvnes FA, Steffenach HA, Murison R, Moser EI, et al (2002) Reduced fear expression after lesions of the ventral hippocampus. Proc Natl Acad Sci U S A 99: 10825–10830. 12149439 10.1073/pnas.152112399 PMC125057

[4] Moser MB, Moser EI, Forrest E, Andersen P, Morris RG (1995) Spatial learning with a minislab in the dorsal hippocampus. Proc Natl Acad Sci U S A 92: 9697–9701. 7568200 10.1073/pnas.92.21.9697 PMC40869

[5] Steffenach HA, Witter M, Moser MB, Moser EI (2005) Spatial memory in the rat requires the dorsolateral band of the entorhinal cortex. Neuron 45: 301–313. 15664181 10.1016/j.neuron.2004.12.044

[6] Dolorfo CL, Amaral DG (1998) Entorhinal cortex of the rat: topographic organization of the cells of origin of the perforant path projection to the dentate gyrus. J Comp Neurol 398: 25–48. 9703026

[7] Swanson LW, Cowan WM (1975) Hippocampo-hypothalamic connections: origin in subicular cortex, not ammon’s horn. Science 189: 303–304. 49928 10.1126/science.49928

[8] Petrovich GD, Canteras NS, Swanson LW (2001) Combinatorial amygdalar inputs to hippocampal domains and hypothalamic behavior systems. Brain Res Brain Res Rev 38: 247–289. 11750934 10.1016/s0165-0173(01)00080-7

[9] Kjelstrup KB, Solstad T, Brun VH, Hafting T, Leutgeb S, et al (2008) Finite scale of spatial representation in the hippocampus. Science 321: 140–143. 18599792 10.1126/science.1157086

[10] de Hoz L, Knox J, Morris RG (2003) Longitudinal axis of the hippocampus: both septal and temporal poles of the hippocampus support water maze spatial learning depending on the training protocol. Hippocampus 13: 587–603. 12921349 10.1002/hipo.10079

[11] Ferbinteanu J, McDonald RJ (2001) Dorsal/ventral hippocampus, fornix, and conditioned place preference. Hippocampus 11: 187–200. 11345125 10.1002/hipo.1036

[12] McDonald RJ, Jones J, Richards B, Hong NS (2006) A double dissociation of dorsal and ventral hippocampal function on a learning and memory task mediated by the dorso-lateral striatum. Eur J Neurosci 24: 1789–1801. 17004942 10.1111/j.1460-9568.2006.05064.x

[13] Bast T, Wilson IA, Witter MP, Morris RG (2009) From rapid place learning to behavioral performance: a key role for the intermediate hippocampus. PLoS Biol 7: e1000089. 19385719 10.1371/journal.pbio.1000089 PMC2671558

[14] Wimer RE, Wimer CC, Winn CJ, Raval NA, Alameddine L, et al (1990) New strains of seizure-prone mice. Brain Res 534: 94–98. 2073601 10.1016/0006-8993(90)90116-s

[15] Rahimi O, Claiborne BJ (2007) Morphological development and maturation of granule neuron dendrites in the rat dentate gyrus. Prog Brain Res 163: 167–181. 17765718 10.1016/S0079-6123(07)63010-6

[16] Chawla MK, Guzowski JF, Ramirez-Amaya V, Lipa P, Hoffman KL, et al (2005) Sparse, environmentally selective expression of Arc RNA in the upper blade of the rodent fascia dentata by brief spatial experience. Hippocampus 15: 579–586. 15920719 10.1002/hipo.20091

[17] Ramirez-Amaya V, Vazdarjanova A, Mikhael D, Rosi S, Worley PF, et al (2005) Spatial exploration-induced Arc mRNA and protein expression: evidence for selective, network-specific reactivation. J Neurosci 25: 1761–1768. 15716412 10.1523/JNEUROSCI.4342-04.2005 PMC6725922

[18] Fevurly RD, Spencer RL (2004) Fos expression is selectively and differentially regulated by endogenous glucocorticoids in the paraventricular nucleus of the hypothalamus and the dentate gyrus. J Neuroendocrinol 16: 970–979. 15667452 10.1111/j.1365-2826.2004.01257.x

[19] Snyder JS, Clifford MA, Jeurling SI, Cameron HA (2012) Complementary activation of hippocampal-cortical subregions and immature neurons following chronic training in single and multiple context versions of the water maze. Behav Brain Res 227: 330–339. 21736899 10.1016/j.bbr.2011.06.025 PMC3212609

[20] VanElzakker M, Fevurly RD, Breindel T, Spencer RL (2008) Environmental novelty is associated with a selective increase in Fos expression in the output elements of the hippocampal formation and the perirhinal cortex. Learn Mem 15: 899–908. 19050162 10.1101/lm.1196508 PMC2632843

[21] Snyder JS, Radik R, Wojtowicz JM, Cameron HA (2009) Anatomical gradients of adult neurogenesis and activity: young neurons in the ventral dentate gyrus are activated by water maze training. Hippocampus 19: 360–370. 19004012 10.1002/hipo.20525 PMC2798730

[22] Jinno S (2011) Topographic differences in adult neurogenesis in the mouse hippocampus: a stereology-based study using endogenous markers. Hippocampus 21: 467–480. 20087889 10.1002/hipo.20762

[23] Piatti VC, Davies-Sala MG, Esposito MS, Mongiat LA, Trinchero MF, et al (2011) The timing for neuronal maturation in the adult hippocampus is modulated by local network activity. J Neurosci 31: 7715–7728. 21613484 10.1523/JNEUROSCI.1380-11.2011 PMC3701257

[24] Banasr M, Soumier A, Hery M, Mocaer E, Daszuta A (2006) Agomelatine, a new antidepressant, induces regional changes in hippocampal neurogenesis. Biol Psychiatry 59: 1087–1096. 16499883 10.1016/j.biopsych.2005.11.025

[25] Olariu A, Cleaver KM, Cameron HA (2007) Decreased neurogenesis in aged rats results from loss of granule cell precursors without lengthening of the cell cycle. J Comp Neurol 501: 659–667. 17278139 10.1002/cne.21268

[26] Choi YS, Cho KO, Kim SY (2007) Asymmetry in enhanced neurogenesis in the rostral dentate gyrus following kainic acid-induced status epilepticus in adult rats. Arch Pharm Res 30: 646–652. 17615686 10.1007/BF02977661

[27] Sahay A, Hen R (2007) Adult hippocampal neurogenesis in depression. Nat Neurosci 10: 1110–1115. 17726477 10.1038/nn1969

[28] Leuner B, Caponiti JM, Gould E (2012) Oxytocin stimulates adult neurogenesis even under conditions of stress and elevated glucocorticoids. Hippocampus 22: 861–868. 21692136 10.1002/hipo.20947 PMC4756590

[29] Tanti A, Rainer Q, Minier F, Surget A, Belzung C (2012) Differential environmental regulation of neurogenesis along the septo-temporal axis of the hippocampus. Neuropharmacology 63: 374–384. 22561281 10.1016/j.neuropharm.2012.04.022

[30] David DJ, Samuels BA, Rainer Q, Wang JW, Marsteller D, et al (2009) Neurogenesis-dependent and -independent effects of fluoxetine in an animal model of anxiety/depression. Neuron 62: 479–493. 19477151 10.1016/j.neuron.2009.04.017 PMC2759281

[31] Snyder JS, Soumier A, Brewer M, Pickel J, Cameron HA (2011) Adult hippocampal neurogenesis buffers stress responses and depressive behaviour. Nature 476: 458–461. 21814201 10.1038/nature10287 PMC3162077

[32] Santarelli L, Saxe M, Gross C, Surget A, Battaglia F, et al (2003) Requirement of hippocampal neurogenesis for the behavioral effects of antidepressants. Science 301: 805–809. 12907793 10.1126/science.1083328

[33] West MJ, Slomianka L, Gundersen HJ (1991) Unbiased stereological estimation of the total number of neurons in thesubdivisions of the rat hippocampus using the optical fractionator. Anat Rec 231: 482–497. 1793176 10.1002/ar.1092310411

[34] Korbo L, Pakkenberg B, Ladefoged O, Gundersen HJ, Arlien-Soborg P, et al (1990) An efficient method for estimating the total number of neurons in rat brain cortex. J Neurosci Methods 31: 93–100. 2181205 10.1016/0165-0270(90)90153-7

[35] Sandoval CJ, Martinez-Claros M, Bello-Medina PC, Perez O, Ramirez-Amaya V (2011) When are new hippocampal neurons, born in the adult brain, integrated into the network that processes spatial information? PLoS ONE 6: e17689. 21408012 10.1371/journal.pone.0017689 PMC3052368

[36] Esposito MS, Piatti VC, Laplagne DA, Morgenstern NA, Ferrari CC, et al (2005) Neuronal differentiation in the adult hippocampus recapitulates embryonic development. J Neurosci 25: 10074–10086. 16267214 10.1523/JNEUROSCI.3114-05.2005 PMC6725804

[37] Zhao C, Teng EM, Summers RG Jr, Ming GL, Gage FH (2006) Distinct morphological stages of dentate granule neuron maturation in the adult mouse hippocampus. J Neurosci 26: 3–11. 16399667 10.1523/JNEUROSCI.3648-05.2006 PMC6674324

[38] Toni N, Laplagne DA, Zhao C, Lombardi G, Ribak CE, et al (2008) Neurons born in the adult dentate gyrus form functional synapses with target cells. Nat Neurosci 11: 901–907. 18622400 10.1038/nn.2156 PMC2572641

[39] Snyder JS, Choe JS, Clifford MA, Jeurling SI, Hurley P, et al (2009) Adult-born hippocampal neurons are more numerous, faster maturing, and more involved in behavior in rats than in mice. J Neurosci 29: 14484–14495. 19923282 10.1523/JNEUROSCI.1768-09.2009 PMC2830901

[40] Kuipers SD, Tiron A, Soule J, Messaoudi E, Trentani A, et al (2009) Selective survival and maturation of adult-born dentate granule cells expressing the immediate early gene Arc/Arg3.1. PLoS ONE 4: e4885. 19290048 10.1371/journal.pone.0004885 PMC2654102

[41] Shepherd JD, Bear MF (2011) New views of Arc, a master regulator of synaptic plasticity. Nat Neurosci 14: 279–284. 21278731 10.1038/nn.2708 PMC8040377

[42] Snyder JS, Glover LR, Sanzone KM, Kamhi JF, Cameron HA (2009) The effects of exercise and stress on the survival and maturation of adult-generated granule cells. Hippocampus 19: 898–906. 19156854 10.1002/hipo.20552 PMC2755652

[43] Kee N, Teixeira CM, Wang AH, Frankland PW (2007) Preferential incorporation of adult-generated granule cells into spatial memory networks in the dentate gyrus. Nat Neurosci 10: 355–362. 17277773 10.1038/nn1847

[44] Racine RJ (1972) Modification of seizure activity by electrical stimulation. II. Motor seizure. Electroencephalogr Clin Neurophysiol 32: 281–294. 4110397 10.1016/0013-4694(72)90177-0

[45] Functional Neurogenesis website. Available: http://www.functionalneurogenesis.com/blog/2011/04/dorsoventral-vs-septotemporal-hippocampus/. Accessed Oct 11, 2012.

[46] Paxinos G, Watson C (1998) The Rat Brain in Stereotaxic Coordinates. San Diego: Academic Press, Inc.

[47] Dayer AG, Ford AA, Cleaver KM, Yassaee M, Cameron HA (2003) Short-term and long-term survival of new neurons in the rat dentate gyrus. J Comp Neurol 460: 563–572. 12717714 10.1002/cne.10675

[48] Cameron HA, Woolley CS, McEwen BS, Gould E (1993) Differentiation of newly born neurons and glia in the dentate gyrus of the adult rat. Neuroscience 56: 337–344. 8247264 10.1016/0306-4522(93)90335-d

[49] Zhao S, Zhou Y, Gross J, Miao P, Qiu L, et al. (2010) Fluorescent labeling of newborn dentate granule cells in GAD67-GFP transgenic mice: a genetic tool for the study of adult neurogenesis. PLoS One 5. 10.1371/journal.pone.0012506 PMC2932690 20824075

[50] Guzowski JF, Miyashita T, Chawla MK, Sanderson J, Maes LI, et al (2006) Recent behavioral history modifies coupling between cell activity and Arc gene transcription in hippocampal CA1 neurons. Proc Natl Acad Sci U S A 103: 1077–1082. 16415163 10.1073/pnas.0505519103 PMC1347968

[51] Shepherd JD, Rumbaugh G, Wu J, Chowdhury S, Plath N, et al (2006) Arc/Arg3.1 mediates homeostatic synaptic scaling of AMPA receptors. Neuron 52: 475–484. 17088213 10.1016/j.neuron.2006.08.034 PMC1764219

[52] Toni N, Teng EM, Bushong EA, Aimone JB, Zhao C, et al (2007) Synapse formation on neurons born in the adult hippocampus. Nat Neurosci 10: 727–734. 17486101 10.1038/nn1908

[53] Ge S, Goh EL, Sailor KA, Kitabatake Y, Ming GL, et al (2006) GABA regulates synaptic integration of newly generated neurons in the adult brain. Nature 439: 589–593. 16341203 10.1038/nature04404 PMC1420640

[54] Jessberger S, Kempermann G (2003) Adult-born hippocampal neurons mature into activity-dependent responsiveness. Eur J Neurosci 18: 2707–2712. 14656319 10.1111/j.1460-9568.2003.02986.x

[55] Stone SS, Teixeira CM, Zaslavsky K, Wheeler AL, Martinez-Canabal A, et al (2011) Functional convergence of developmentally and adult-generated granule cells in dentate gyrus circuits supporting hippocampus-dependent memory. Hippocampus 21: 1348–1362. 20824726 10.1002/hipo.20845

[56] Dong HW, Swanson LW, Chen L, Fanselow MS, Toga AW (2009) Genomic-anatomic evidence for distinct functional domains in hippocampal field CA1. Proc Natl Acad Sci U S A 106: 11794–11799. 19561297 10.1073/pnas.0812608106 PMC2710698

[57] Hortnagl H, Berger ML, Sperk G, Pifl C (1991) Regional heterogeneity in the distribution of neurotransmitter markers in the rat hippocampus. Neuroscience 45: 261–272. 1684835 10.1016/0306-4522(91)90224-c

[58] Segal M, Richter-Levin G, Maggio N (2010) Stress-induced dynamic routing of hippocampal connectivity: a hypothesis. Hippocampus 20: 1332–1338. 20082290 10.1002/hipo.20751

[59] Hight K, Hallett H, Churchill L, De A, Boucher A, et al (2010) Time of day differences in the number of cytokine-, neurotrophin- and NeuN-immunoreactive cells in the rat somatosensory or visual cortex. Brain Res 1337: 32–40. 20398636 10.1016/j.brainres.2010.04.012 PMC2892412

[60] Satvat E, Schmidt B, Argraves M, Marrone DF, Markus EJ (2011) Changes in task demands alter the pattern of zif268 expression in the dentate gyrus. J Neurosci 31: 7163–7167. 21562279 10.1523/JNEUROSCI.0094-11.2011 PMC6703202

[61] Snyder JS, Ramchand P, Rabbett S, Radik R, Wojtowicz JM, et al (2011) Septo-temporal gradients of neurogenesis and activity in 13-month-old rats. Neurobiol Aging 32: 1149–1156. 19632743 10.1016/j.neurobiolaging.2009.05.022 PMC2889161

[62] Cameron HA, Tanapat P, Gould E (1998) Adrenal steroids and N-methyl-D-aspartate receptor activation regulate neurogenesis in the dentate gyrus of adult rats through a common pathway. Neuroscience 82: 349–354. 9466447 10.1016/s0306-4522(97)00303-5

[63] Tanapat P, Hastings NB, Rydel TA, Galea LA, Gould E (2001) Exposure to fox odor inhibits cell proliferation in the hippocampus of adult rats via an adrenal hormone-dependent mechanism. J Comp Neurol 437: 496–504. 11503148 10.1002/cne.1297

[64] Tanapat P, Hastings NB, Reeves AJ, Gould E (1999) Estrogen stimulates a transient increase in the number of new neurons in the dentate gyrus of the adult female rat. J Neurosci 19: 5792–5801. 10407020 10.1523/JNEUROSCI.19-14-05792.1999 PMC6783062

[65] Dranovsky A, Picchini AM, Moadel T, Sisti AC, Yamada A, et al (2011) Experience dictates stem cell fate in the adult hippocampus. Neuron 70: 908–923. 21658584 10.1016/j.neuron.2011.05.022 PMC3124009

[66] Snyder JS, Cameron HA (2012) Could adult hippocampal neurogenesis be relevant for human behavior? Behav Brain Res 227: 384–390. 21736900 10.1016/j.bbr.2011.06.024 PMC3210392

[67] Aimone JB, Wiles J, Gage FH (2009) Computational influence of adult neurogenesis on memory encoding. Neuron 61: 187–202. 19186162 10.1016/j.neuron.2008.11.026 PMC2670434

